# Optimization of Sodium Iodide-Based Root Filling Material for Clinical Applications: Enhancing Physicochemical Properties

**DOI:** 10.3390/pharmaceutics16081031

**Published:** 2024-08-02

**Authors:** Hye-Shin Park, Yu-Jin Kim, Soo-Jin Chang, Hae-Hyoung Lee, Mi-Ran Han, Joon-Haeng Lee, Jong-Soo Kim, Jong-Bin Kim, Ji-Sun Shin, Jung-Hwan Lee

**Affiliations:** 1 Department of Pediatric Dentistry, College of Dentistry, Dankook University, 119 Dandae-ro, Cheonan 31116, Republic of Korea; gptls1209@naver.com (H.-S.P.); changsoo713@gmail.com (S.-J.C.); miraneee@dankook.ac.kr (M.-R.H.); haeng119@naver.com (J.-H.L.); jskim@dku.edu (J.-S.K.); pedoshin@dankook.ac.kr (J.-S.S.); 2 Department of Biomaterials Science, College of Dentistry, Dankook University, 119 Dandae-ro, Cheonan 31116, Republic of Korea; yujin10316426@gmail.com (Y.-J.K.); haelee@dku.edu (H.-H.L.); 3 Institute of Tissue Regeneration Engineering (ITREN), Dankook University, 119 Dandae-ro, Cheonan 31116, Republic of Korea; 4 Department of Nanobiomedical Science & BK21 PLUS NBM Global Research Center for Regenerative Medicine, Dankook University, 119 Dandae-ro, Cheonan 31116, Republic of Korea; 5 UCL Eastman-Korea Dental Medicine Innovation Centre, Dankook University, 119 Dandae-ro, Cheonan 31116, Republic of Korea; 6 Cell & Matter Institute, Dankook University, 119 Dandae-ro, Cheonan 31116, Republic of Korea; 7 Mechanobiology Dental Medicine Research Center, Dankook University, 119 Dand-ro, Cheonan 31116, Republic of Korea

**Keywords:** sodium iodide, root filling material, vitapex, lanolin, solubility, radiopacity, injectability, viscosity, antibacterial test

## Abstract

Premature loss of root canal-treated primary teeth has long been a concern in dentistry. To address this, researchers developed a sodium iodide-based root canal-filling material as an alternative to traditional iodoform-based materials. The goal of this study was to improve the physicochemical properties of the sodium iodide-based material to meet clinical use standards. To resolve high solubility issues in the initial formulation, researchers adjusted component ratios and added new ingredients, resulting in a new paste called L5. This study compared L5 with L0 (identical composition minus lanolin) and Vitapex as controls, conducting physicochemical and antibacterial tests. Results showed that L5 met all ISO 6876 standards, demonstrated easier injection and irrigation properties than Vitapex, and exhibited comparable antibacterial efficacy to Vitapex, which is currently used clinically. The researchers conclude that if biological stability is further verified, L5 could potentially be presented as a new option for root canal-filling materials in primary teeth.

## 1. Introduction

Numerous case studies have documented premature exfoliation of deciduous teeth following root canal therapy, attributed to accelerated root resorption [1,2,3,4,5]. Petel et al. [6] assessed the cytotoxicity of iodoform-containing endodontic materials on RAW macrophages and RKO epithelial cells. Compared to calcium hydroxide-based materials, higher cytotoxicity and potential for cystic changes suggested iodoform may induce cellular stress. Moskovitz et al. [7] found greater root resorption in deciduous teeth obturated with Vitapex^®^ compared with 3Mix paste at 6 and 12 months, radiographically. Moreover, Nakornchai et al. [8] revealed more resorption in endodontically treated deciduous teeth relative to contralateral untreated teeth, confirming root canal treatment can hasten physiological resorption.

**Figure 1:**
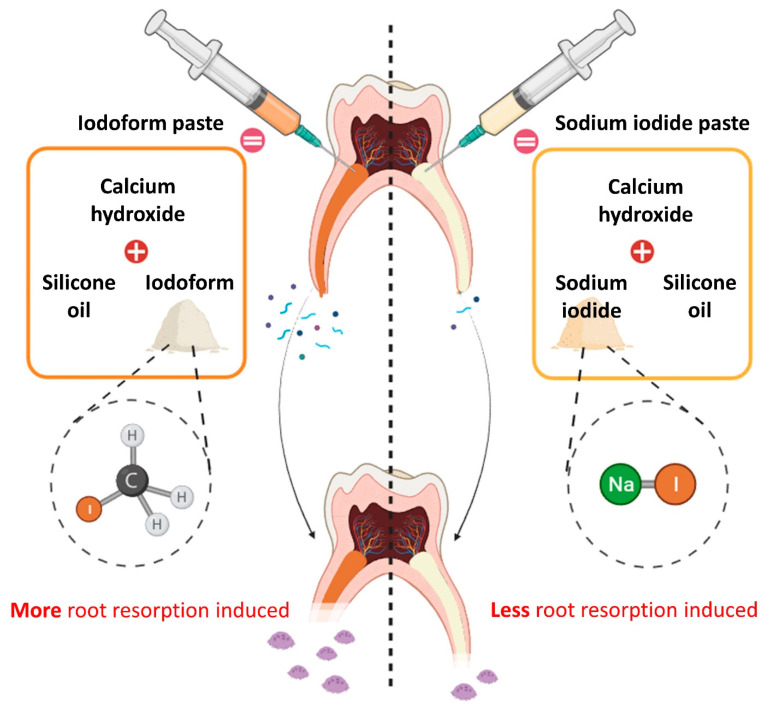
Research schematic diagram. There has been research indicating that iodoform-based root canal-filling material promotes root resorption in deciduous teeth [1,2,3,4,5]. Previous studies have suggested that substituting iodoform with sodium iodide could reduce the increased activity of osteoclasts, ultimately resulting in less root resorption [12]. This study was designed to introduce a new root canal-filling material clinically.

Vitapex^®^ is composed of calcium hydroxide, iodoform, silicone oil, and other components not disclosed by the manufacturer [9]. Based on research suggesting that iodoform is associated with root resorption, our laboratory conducted research on potential components that could replace iodoform [7]. Since the combination of iodoform and calcium hydroxide enhances antibacterial effects [10], we investigated other substitute substances containing iodide groups. During this research, we selected sodium iodide, which is used in thyroid cancer treatments, as a potential replacement cause of its biocompatibility [11]. In 2022, Choi et al. [12] developed a new primary tooth-obturating material substituting sodium iodide for iodoform in Vitapex’s calcium hydroxide and silicone oil formulation. As NFATc1 regulates osteoclast maturation and c-Fos controls NFATc1, these transcription factors mediate RANKL-induced osteoclast activation [13]. Additionally, tartrate-resistant acid phosphatase (TRAP) and cathepsin K, which are osteoclastogenesis-related genes, were used as markers to confirm the degradation of bone mineral and collagen matrices [14]. The study by Choi et al. showed that sodium iodide-based pastes downregulated TRAP, cathepsin K, NFATc1, and c-Fos compared with iodoform-based pastes, suggesting reduced osteoclastogenesis and root resorption potential [12]. (Figure 1). In 2023, Chang et al. [15] aimed to enhance the injectability of a silicone oil-based paste by testing various oil vehicles. While low-density silicone oil optimized injectability, the solubility of the developed paste did not meet the ISO 6876 standard (flow ≥ 17 mm, film thickness ≤ 50 μm, radiopacity ≥ 3 mm aluminum wedge, solubility ≤ 3%) [16], making it unsuitable for clinical use. If the solubility is higher than the standard, the paste would dissolve beyond the root apex, preventing it from functioning as a root canal-filling material [17].

This study’s objective was to optimize the physicochemical properties of the sodium iodide-based obturating material, particularly addressing the prior paste’s high solubility. Strategies include (1) reducing calcium hydroxide and sodium iodide percentages [18]; (2) increasing silicone oil content [19]; and (3) supplementing with PEG, xanthan gum, and lanolin additives [20,21,22]. By applying these approaches, we determined an optimal paste composition suitable for clinical applications.

## 2. Material and Methods

### 2.1. Preparation for Sample

In this study, calcium hydroxide (Sigma-Aldrich, Burlington, MA, USA), sodium iodide (AlfaAesar, Heysham, UK), and low-density silicone oil (Shin-Etsu Silicone KF-96 350 cst, Shin-Etsu Chemical Co., Tokyo, Japan) were employed. To determine the formulation of an injectable paste with suitable solubility, the solubility of multiple injectable pastes with varying proportions of each material was evaluated. Moreover, supplementary ingredients such as polyethylene glycol (PEG) (Deajung, Gyeonggi-do, Republic of Korea), xanthan gum (Jungbunzlauer, Wien, Austria), and lanolin (Deajung, Gyeonggi-do, Republic of Korea) were incorporated. The injectable pastes were prepared by blending the components on a glass surface using a spatula under sterile conditions. The compositions of all experimental groups are outlined in Table 1.

### 2.2. Physicochemical Characteristics of Samples

Solubility, flow, film thickness, and radiopacity tests were performed on the control and experimental groups according to the International Standard Organization (ISO) 6876:2012 (Dentistry—Root Canal Sealing Materials). During the experimental process to reduce solubility, the L5 group containing 28.75% calcium hydroxide, 28.75% sodium iodide, and 5% lanolin exhibited adequate solubility. Based on the solubility test results, the L5 group that met the ISO solubility specification, and the L0 group, which has the same composition as L5 except lanolin, were selected along with the Vitapex^®^ control group to proceed with subsequent experiments. All experiments were carried out at room temperature (23 ± 2 °C) and 5% relative humidity unless otherwise specified. All experiments were performed in triplicates and the mean values were used for comparisons.

### 2.3. Solubility

A total of 2 molds, each with an internal diameter of approximately 20 ± 1 mm and a height of around 1.5 ± 0.1 mm, were filled with the injectable paste. The weight of each specimen was measured to the nearest 0.001 g and recorded as the initial weight (I0). The mold-filled sealers were placed in Petri dishes, with one surface exposed to 50 mL (±1 mL) of distilled water added to each dish. These samples were then stored for 24 h in a climate-controlled chamber maintained at 37 °C (±1 °C) temperature and 95% relative humidity. After this period, the solution was filtered through a filter paper, and the filtered solution was dried in an oven set at 80 ± 2 °C. The weight of the empty beaker was recorded as I1, and the weight of the beaker with the dried residue was recorded as I2. The solubility percentage was calculated by subtracting I1 from I2, dividing the result by the initial sample weight (I0), and multiplying by 100.

$$Solubility=\frac{Final\;weight\;of\;beaker\left(I2\right)-initial\;weight\;of\;beaker\;(I1)}{initial\;weight\;of\;sample\;(I0)}\times 100\;(\%)$$

#### 2.3.1. Flow

Two glass plates with dimensions of 40 mm × 40 mm × 5 mm and a mass of 20 g each were prepared. A quantity of 0.05 ± 0.005 mL of the sealing material was placed at the center of one glass plate. After a period of 180 ± 5 s, an identical glass plate was positioned over the sealing material, with a weight of 100 g placed on top. At 600 s from the start of mixing, the weight was removed, and the minimum and maximum diameters of the circular sealing material were measured. If the difference between these diameters was within 1 mm, the average value was calculated and recorded as the flow value.

#### 2.3.2. Film Thickness

Two glass plates were prepared, each having a contact area of 200 ± 25 mm^2^ and a minimum thickness of 5 mm. The combined initial thickness of the overlapping plates was measured with an accuracy of 1 μm. A small quantity of the sealing material was applied at the center of one plate, and the other plate was placed on top. After a period of 180 ± 10 s, this assembly was loaded into a loading device, and a vertical force of 150 ± 3 N was applied. At 10 min from the start of mixing, it was verified that the sealing material had spread across the entire surface of the glass plate before measuring the combined thickness of the two plates with the sealing material in between. The film thickness was then calculated by subtracting the initial combined thickness of the two glass plates without the sealing material from this final measurement.

#### 2.3.3. Optical Images

To evaluate and compare the surface characteristics of the three root canal-filling injectable pastes, a small quantity of 0.01 mL from each injectable paste was applied onto separate glass plates. Optical microscopic images of these samples were then captured using a 25× magnification microscope (Model S39A, Microscopes Instrument, Suwon, Republic of Korea).

#### 2.3.4. Radiopacity

A mold made of radiolucent materials like plastic or paper was prepared, having an internal diameter of 10 mm and a height of 1.00 ± 0.01 mm. An aluminum step wedge, 50 mm in length and 20 mm in width, composed of aluminum with a purity of at least 98% and containing no more than 0.1% copper and 1.05% iron, was used along with the specimen-filled mold. These were placed on an X-ray film (Kodak Insight, Rochester, NY, USA). X-ray images were acquired using a Kodak-2200 X-ray machine (Kodak Insight, Rochester, NY, USA) under the following conditions: 7 mA current, 70 kV voltage, 0.3 s exposure time, and a source-to-film distance of 300 mm. The obtained X-ray images were analyzed using ImageJ version 1.53a grayscale software (National Institutes of Health, Bethesda, MD, USA). The radiopacity of the specimens was compared to different thickness steps of the aluminum wedge. The visual images were quantified numerically using the formula described by Hungaro Duarte et al. [23] to enable objective comparisons.

#### 2.3.5. Injectability

To evaluate the injectability, a mold designed to accommodate a 1 mL syringe was fabricated and utilized for conducting the experiments. The injection force was measured by applying a controlled force to each injectable paste at a rate of 0.05 mm/sec until the paste was extruded from the needle tip [24].

#### 2.3.6. Viscosity

To measure the viscosity, 0.3 mL of the injectable paste was evenly distributed onto a Peltier plate with a diameter of 60.0 mm. The viscosity analyses were performed using a Discovery HR-1 rheometer (TRIOS, TA Instruments, New Castle, DE, USA). The top aluminum plate was lowered by 500 μm, and any excess material was removed. All the viscosity tests were conducted at room temperature, with the frequency ranging from 0.05 to 1000 Hz [25].

#### 2.3.7. Extraction Test

For the experiments involving pH variation and ion release, approximately 1 ± 0.1 g of the injectable paste was stored in 5500 μL of distilled water and placed in a shaking incubator maintained at 37 ± 1 °C for 24 h. After 24 h, the solution was filtered through a 0.22 μm filter (Corning Incorporated, Corning, NY, USA). The filtered solution was then used for inductively coupled plasma–atomic emission spectrometry (ICP-AES) analysis using an Optima 8300 instrument (PerkinElmer, Waltham, MA, USA), ion chromatography, and pH testing. An Orion VERSA Star Pro pH meter (Thermo Fisher Scientific, Waltham, MA, USA), calibrated with pH 4.01, 7.00, and 10.01 buffer solutions, was employed for the pH measurements, using the same solution utilized for the ion release experiments [26]. The pH and ion release tests were conducted in triplicates, and the mean and standard deviation values were reported.

#### 2.3.8. Endodontic Treatment for Resin Tooth

(1) Root-filling ability evaluation

To evaluate the clinical handling of the injectable pastes as primary root canal fillings, conventional root canal treatment was performed on 12 mandibular premolar resin teeth models. The working length was determined by taking a diagnostic radiograph with a #15 K-file inserted into the root canal until its tip was visible 1 mm short of the radiographic apex. Root canal shaping was carried out using K-files up to size #35, with irrigation using normal saline and sodium hypochlorite solutions [27,28]. Paper points were used to dry the root canals before filling them with the injectable paste delivered via syringes. The paste was injected until it extruded through the canal orifice, and then the syringes were carefully withdrawn. The access cavities were also filled with the injectable paste for uniform evaluation [29]. Post-operative periapical radiographs were taken to assess the quality of the root canal fillings.

(2) Removability of Pastes

To assess the removability of the injectable pastes, resin teeth that had been filled with the injectable paste were subjected to ultrasonic removal procedures using an ultrasonic scaler. The tip of the ultrasonic scaler was positioned at the level of the access cavity orifice and activated in a static mode for 1 min to ultrasonically remove the injectable paste [30]. Periapical radiographs were taken after the removal process to examine the remaining root canal-filling material.

### 2.4. Antibacterial Test

Enterococcus faecalis (*E. faecalis*, ATCC 19433) was obtained from the American Type Culture Collection (ATCC) in Manassas, VA, USA. The bacterial colonies were streaked onto brain heart infusion agar (BHIA; BD Difco, Franklin Lakes, NJ, USA) plates and incubated at 37 °C for 24 h. A single colony was then transferred to brain heart infusion broth (BHIB; BD Difco, Franklin Lakes, NJ, USA) and incubated at 37 °C for 18 h.

In a culture plate, 0.3 g of the specimen material was placed in each well, and 3 mL of an *E. faecalis* suspension containing 3 × 10^6^ CFU/mL was added on top. The positive control group did not contain any specimen material (bacteria only). The plates were then incubated in a 37 °C, 5% CO_2_ incubator for 1 h.

After 1 h of direct contact between the specimen, bacteria, and medium in each well, serial dilutions were performed, and the diluted samples were plated. All plates were incubated at 37 °C for 24 h. Following incubation, bacterial colonies were counted and recorded.

### 2.5. Statistical Analysis

The data were presented as mean ± standard deviation (SD) values and analyzed using SPSS software version 26.0 (SPSS Inc., Chicago, IL, USA). The Kruskal–Wallis test, along with Dunnett’s multiple comparison test, was employed to evaluate the distribution of data within groups. The level of significance was set at α = 0.05, and the notation ‘ns’ was used to indicate no statistical significance.

## 3. Results

### 3.1. Physicochemical Properties

#### 3.1.1. Solubility

**Figure 2:**
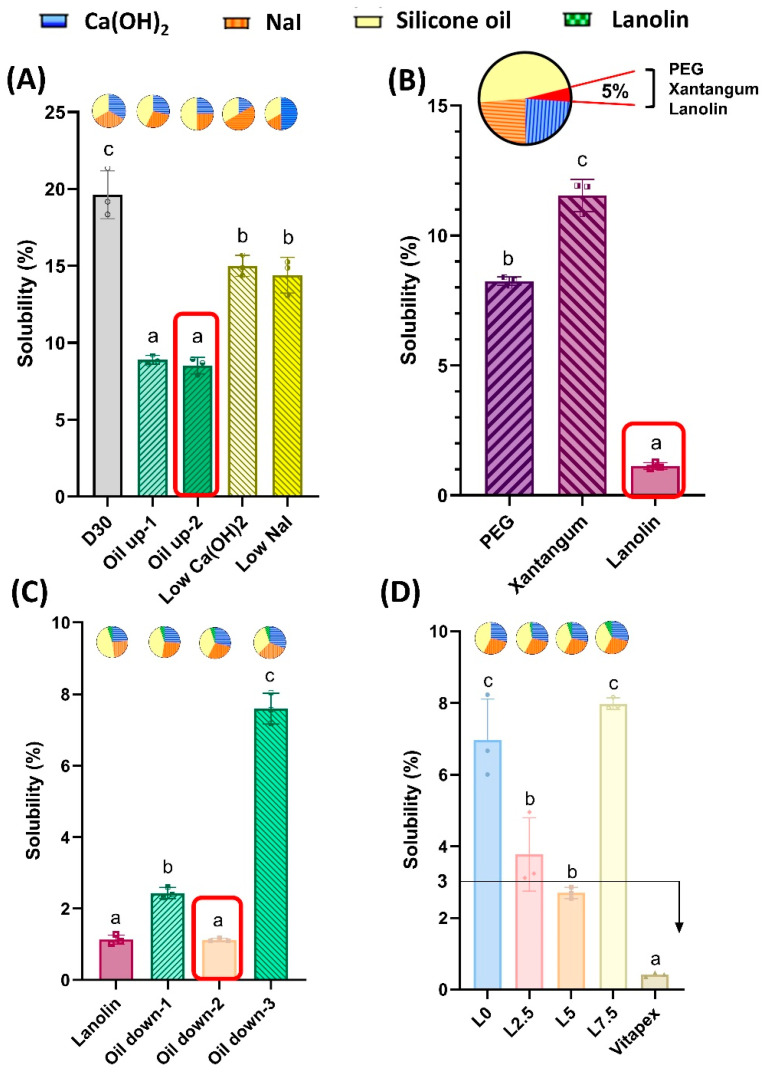
To reduce solubility, a four-step process was undertaken as follows. (**A**) Modification of the material ratios of calcium hydroxide, sodium iodide, and silicone oil in the existing D30 paste. The oil up-2 group, exhibiting the most stable solubility, was selected. (**B**) Observation of solubility improvement effects through additives. The lanolin group, showing a decrease in solubility, was chosen. (**C**) Experimentation to reduce the oil content. Subsequently, the oil down-2 group, characterized by the most stable solubility and a lower oil content, was selected. (**D**) Observation of solubility changes with varying amounts of lanolin. The L5 group, demonstrating the most stable solubility, showed a solubility lower than 3%. Statistically significant differences are presented in different superscript letters (*p* < 0.05).

In the previously developed D30 paste formulation [30], varying the ratios of calcium hydroxide, sodium iodide, and silicone oil components demonstrated that the oil up-2 group, containing the highest percentage of silicone oil, exhibited the lowest solubility (Figure 2A).

Adopting the oil up-2 group formulation and substituting 5% of the silicone oil with three alternative additives—polyethylene glycol, xanthan gum, and lanolin—revealed that only the lanolin additive was effective in reducing the solubility (Figure 2B).

Evaluation of formulations with overall lower oil percentages based on the lanolin-containing group (oil down-1, 2, and 3 groups) showed that the oil down-2 group exhibited the most stable solubility (Figure 2C).

Experiments investigating the effect of different lanolin addition amounts on solubility demonstrated that the formulation containing 5% lanolin (L5 group) met the ISO 6876 standard requirement of ≤3% solubility (Figure 2D).

#### 3.1.2. Flow

**Figure 3:**
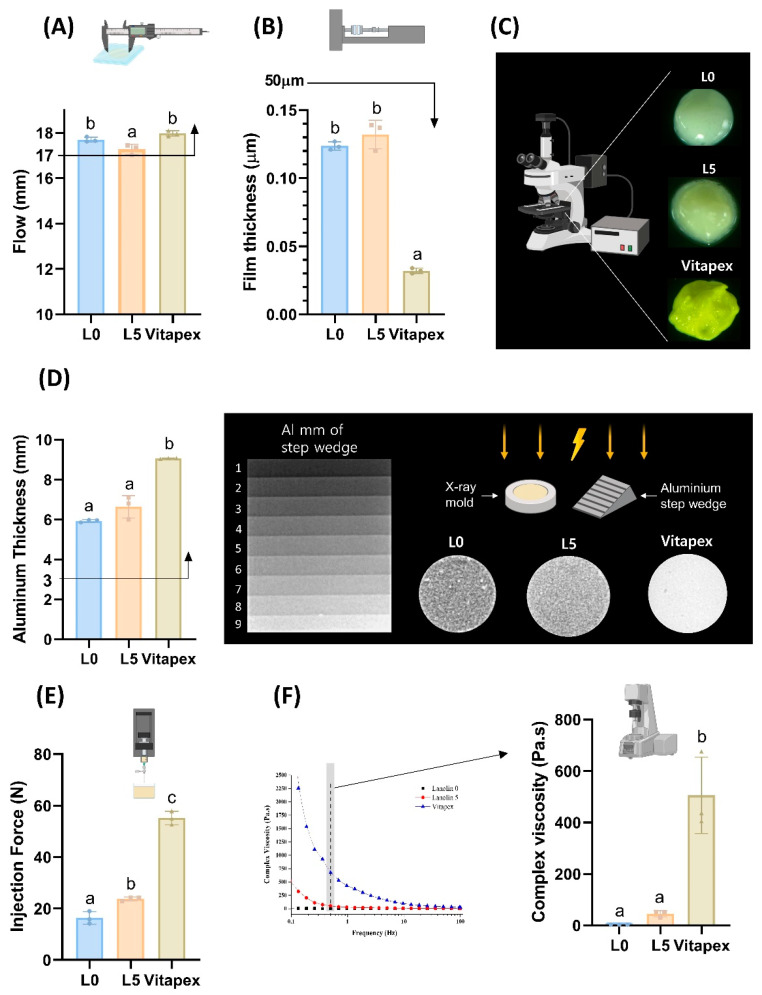
Physical test for paste. Physical tests were conducted on the L0, L5, and Vitapex 3 groups. All the tests followed by ISO 6876. (**A**) The flowability test demonstrated that all three groups satisfied the ISO 6876 standard of 17 mm or more. (**B**) The film thickness test demonstrated that all 3 groups met the ISO 6876 criterion of 50 μm or less. (**C**) Optical image of 3 pastes, (**D**) radiopacity test demonstrated that all 3 groups fulfilled the ISO 6876 standard of an aluminum thickness of 3 mm or more. (**E**) The injectability test demonstrated that Vitapex, L5, and L0 required increasing injection force in that order. (**F**) The viscosity test demonstrated that there was no significant difference between L0 and L5, while Vitapex exhibited significantly higher viscosity. Statistically significant differences are presented in different superscript letters (*p* < 0.05).

All three experimental groups—L0, L5, and Vitapex^®^—met the ISO 6876 standard for a flow of ≥17 mm, with a significant difference observed between L5 and Vitapex^®^ (Figure 3A).

#### 3.1.3. Film Thickness

All experimental groups satisfied the ISO 6876 requirement of less than a 50 μm particle size, with significant differences noted between the Vitapex^®^, L0, and L5 groups (Figure 3B).

#### 3.1.4. Optical Images

Optical imaging revealed that the L5 groups exhibited a smoother surface texture compared to Vitapex^®^ (Figure 3C).

#### 3.1.5. Radiopacity

In radiopacity testing, all groups met the ISO 6876 standard for a 3 mm aluminum wedge. Significant differences were observed between the Vitapex^®^, L0, and L5 groups (Figure 3D).

#### 3.1.6. Injectability

Injectability testing showed that more force was required in the order of Vitapex^®^, L5, and L0 (Figure 3E), with significant differences between groups.

#### 3.1.7. Viscosity

Viscosity testing indicated that Vitapex^®^ had significantly higher viscosity compared to the L0 and L5 groups (Figure 3F).

#### 3.1.8. Extraction Test

**Figure 4:**
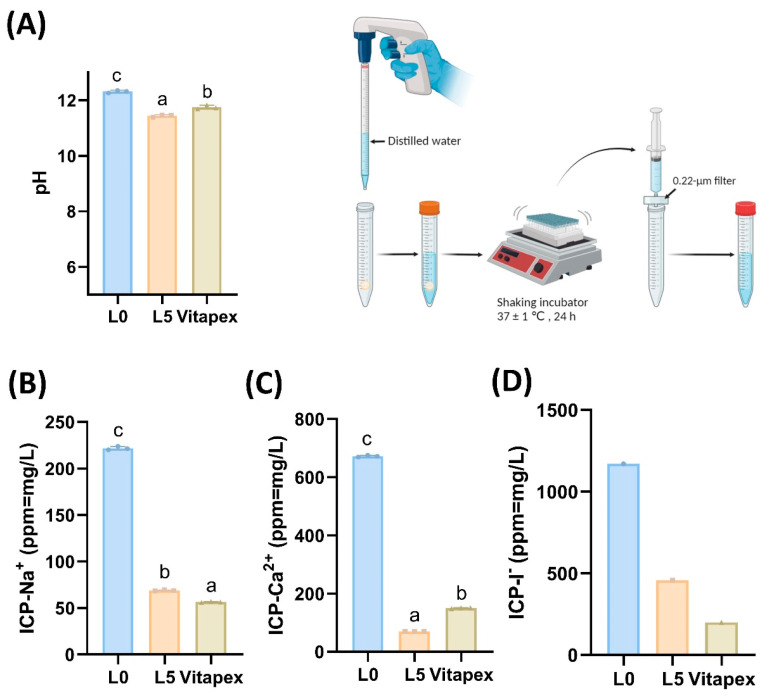
Chemical test for paste. Chemical tests conducted on the L0, L5, and Vitapex groups. After leaching each sample for 24 h, pH testing and ion chromatographic tests for Na^+^, Ca^2+^, and I^−^ ion were performed. (**A**) The pH test indicated that the L0 group, exhibiting the highest solubility, had the highest pH. (**B**) ICP-Na^+^ test results indicated that the L5 group released more Na^+^ ions than Vitapex. (**C**) ICP-Ca^2+^ test results indicated that the Vitapex group released more Ca^2+^ ions than the L5 group. (**D**) ICP-I^−^ test results indicated that iodide ions were released more in the L5 group. Statistically significant differences are presented in different letters (a–c) (*p* < 0.05).

In pH testing of extraction solutions, values decreased in the order of L0, Vitapex^®^, and L5 (Figure 4A), with significant differences between groups. Calcium ion chromatography results corresponded to the pH results (Figure 4C). Sodium ion chromatography revealed a decreasing order of L0, L5, and Vitapex^®^, and iodine ions showed similar trends (Figure 4B,D).

#### 3.1.9. Endodontic Treatment for Resin Tooth

(1) Root-filling ability evaluation

**Figure 5:**
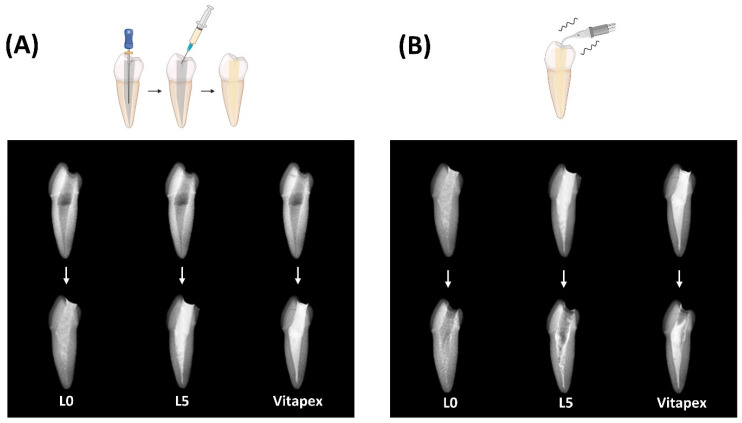
Root canal-filling ability and paste removal test for paste. (**A**) Radiographs of each tooth after root canal filling with each paste following the preparation of the resin tooth. (**B**) Radiographs of each tooth after washing with an ultra scaler for 10 s under the same intensity and frequency conditions to confirm paste removability.

Radiographic imaging of conventionally treated artificial teeth suggested similar abilities of the L0, L5, and Vitapex^®^ groups to reach the apex (Figure 5A).

(2) Removability of paste

With ultrasonic irrigation, Vitapex^®^ showed almost no washout, while more L0 was washed out compared to L5 (Figure 5B).

### 3.2. Antibacterial Test for Paste

**Figure 6:**
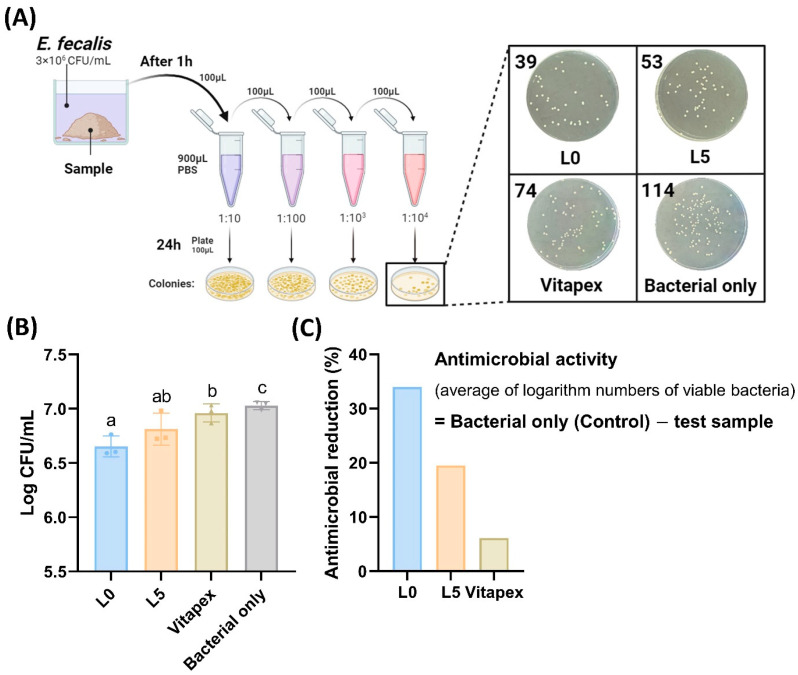
Antibacterial test for paste. (**A**) The medium in which the specimen and bacteria were in direct contact with each well for 1 h was plated by serial dilutions. All plates were incubated at 37 °C for 24 h. After incubation, bacterial colonies were counted and recorded. (**B**) Log CFU/mL of each specimen. The L5 group has no significant difference with Vitapex. (**C**) The percentage of antimicrobial reduction is presented. The calculation formula is presented on the right side. Statistically significant differences are presented in different superscript letters (*p* < 0.05).

Antibacterial testing demonstrated a decreasing antimicrobial reduction in the order of L0, L5, and Vitapex^®^ (Figure 6C). No significant difference in antibacterial efficacy was observed between L5 and Vitapex^®^ (Figure 6B).

## 4. Discussion

To prevent the premature exfoliation of primary teeth that have undergone root canal treatment, we aimed to develop a sodium iodide-based paste as an alternative to the existing iodoform-based paste. In order to improve the physicochemical properties of this new paste, we adjusted the ratios of its components and added a new material called lanolin. In an effort to prevent the early loss of primary teeth that have undergone root canal treatment, successful root canal treatment of primary teeth is a crucial solution [31,32]. Primary teeth afflicted with asymptomatic irreversible pulpitis are able to be maintained by having a proper pulpectomy procedure performed on them [33], and successful primary tooth root canal treatment prevents premature exfoliation and reduces the impact on the succeeding permanent dentition [34]. Achieving successful treatment requires the dentist to make an accurate diagnosis, develop a clear treatment plan, follow proper procedural steps, and select suitable materials [35]. The ideal root canal-filling material for primary teeth should possess the following characteristics: it should resorb at a rate matching the physiological root resorption process without obstructing the eruption of permanent teeth, be easily placed into and removed from the canal, exhibit antibacterial properties, not cause discoloration of the tooth, be radiopaque, adhere well to the canal walls, and be non-harmful to the periapical tissues and developing permanent tooth buds [36].

Zinc oxide eugenol (ZOE) has conventionally been used as a root canal-filling paste for primary teeth after pulpectomy [37]. However, some disadvantages like slow resorption rate [38], periapical tissue irritation [39], and potential for bone and cementum necrosis [40], and deflection of underlying permanent tooth buds [34] have been observed. In 1920, calcium hydroxide (Ca(OH)_2_) paste was introduced by Hermann and widely used [10]. Iodoform was later added by Nurko et al. [41], which increased the antibacterial effect, improved healing, and enhanced resorbability when extruded beyond the apex [42]. High success rates of 84–100% were reported for Ca(OH)_2_/iodoform pastes [42]. For these reasons, Ca(OH)_2_/iodoform pastes like the well-known Vitapex^®^ have long been used as primary tooth root canal-filling materials [18,43,44].

Despite iodoform-based pastes like Vitapex^®^ being less biocompatible than zinc oxide eugenol (ZOE) pastes [45], they have been utilized due to their higher degree of resorption when overfilled beyond the apex in comparison to ZOE and Ca(OH)_2_ pastes [46]. When extruded beyond the tooth root apex, the paste should resorb without causing external root resorption. Therefore, this study aimed to develop a paste containing calcium hydroxide and iodine ions that readily resorb if exposed beyond the apex without leading to external root resorption [27,47,48].

Calcium hydroxide has high antibacterial properties and is a major component of dental root canal medicaments [49,50,51]. Among root-filling materials based on calcium hydroxide, the combinations containing iodoform were shown to possess the highest antibacterial efficacy [52]. This potent effect is possible because iodine ions released from iodoform precipitate proteins and oxidize essential enzymes [53]. Therefore, this study’s purpose was to develop a calcium hydroxide and iodine ion-containing paste that could readily resorb when extruded beyond the apex.

Two methods were adopted to reduce solubility: increasing the silicone oil proportion and adding lanolin. Lanolin is a component of Maisto’s paste, a primary tooth root-filling material [31]. Maisto’s paste, combining iodoform and ZOE, has shown higher success rates than ZOE alone [54]. In comparison to ZOE sealers, Maisto’s paste is regarded as more biocompatible [53] and is considered one of the more biocompatible root-filling materials containing iodoform [55]. When utilized for root canal-filling procedures, high clinical and radiographic success rates of 91.5% and 88.3%, respectively, were exhibited by Maisto’s paste [56]. Furthermore, any excess paste that was extruded was resorbed within 3 months, accompanied by 93% bone regeneration [57]. Successful apical closure was observed 6 months after treatment, indicating clinically stable results [53,58]. Based on these findings, lanolin was selected as an additive for the new paste formulation.

Lanolin is widely utilized as a vehicle in cosmetics, pharmaceuticals, etc. [59]. It is famously known as a major ingredient in ointments that are applied to the nipple for treating nipple trauma in breastfeeding patients [60]. Recent research has also led to the development of lanolin into capsule membranes [61], and its use as a carrier to directly inject glyceryl trinitrate for modulating blood flow has been studied [62], suggesting that lanolin is a biocompatible material.

Physical tests were conducted on three experimental groups of injectable pastes: (1) the L5 injectable paste, which exhibited stable solubility; (2) the L0 injectable paste without lanolin but containing the same ratios of calcium hydroxide and sodium iodide as L5; and (3) the conventionally used Vitapex^®^ paste. Through a series of processes aimed at reducing solubility, we were able to decrease the average solubility of D30 from 19.63% to 2.69% in L5. Although we found a component with an even lower solubility of 1.13%, L5 was expected to have the most suitable physicochemical properties and antibacterial activity as a result of finding the optimal ratio of calcium hydroxide and sodium iodide. All three groups were found to meet the ISO 6876 criteria at flow, film thickness, and radiopacity (Figure 3). Additionally, in comparison to Vitapex^®^, easier injectability was observed with the L0 and L5 groups, which is expected to enable easier root canal application (Figure 3E,F). When used to fill artificial resin teeth, proper filling to the apex was demonstrated by all three groups: L0, L5, and Vitapex^®^. With the use of ultrasonic irrigation, easy cleanliness was observed for the L0 and L5 groups (Figure 5). Less cleanliness was observed for the Vitapex^®^ group, which means it is more difficult in terms of irrigation when we need to administer re-endodontic treatment. Root-filling materials that can be easily cleaned can greatly facilitate retreatment procedures. Furthermore, due to its ease of removal, the potential use of this material as a root canal sealer for permanent teeth could be explored.

The chemical tests revealed that the L0 and L5 groups released more ions compared to Vitapex^®^ because of their higher solubility (Figure 4B–D). Since the main antibacterial actions of root canal-filling pastes come from iodine ions and calcium hydroxide, the higher antibacterial effectiveness of L0 over Vitapex^®^ can be explained by the same principle (Figure 6B,C). In the antibacterial tests, the L5 group demonstrated antibacterial efficacy that was equal to or better than Vitapex^®^ (Figure 6).

The results confirmed that the L5 formulation possesses appropriate physicochemical properties to be utilized clinically, as well as powerful antibacterial effects. However, before L5 can be considered for actual clinical use on patients, more comprehensive biocompatibility evaluations need to be conducted [63]. While biocompatibility has been confirmed for the previously developed paste [15], and the newly added material, lanolin, is known to be biocompatible, it is necessary to verify the synergistic effects of these components when combined. Further studies, including cytotoxicity tests and animal experiments, should be conducted to confirm the overall biocompatibility of the new formulation. After these comprehensive biocompatibility assessments have been completed and yielded positive results, clinical use of this material be considered.

## 5. Conclusions

To overcome the limitations of existing root canal-filling materials for primary teeth, a series of processes were undertaken to develop a clinically viable sodium iodide-based root canal-filling material, resulting in the creation of a paste called L5. L5 contains 5% lanolin. Physicochemical experiments confirmed that L5 meets all ISO 6876 standards, demonstrating its potential for clinical use. It was found that sodium iodide-based root canal-filling materials activate osteoclasts less than iodoform-based materials, potentially leading to less external root resorption. This suggests that when applied clinically, sodium iodide-based materials like L5 may prevent premature exfoliation of primary teeth and reduce subsequent orthodontic problems. The incorporation of lanolin, known for its biocompatible properties, may further enhance the overall performance of L5. However, despite these promising results, additional biocompatibility studies, including cytotoxicity tests and animal experiments, are necessary to fully evaluate the safety profile of L5 before it can be considered for clinical application in patients.

## Figures and Tables

**Table 1:** Composition of the tested pastes.

		Ca(OH)_2_	NaI (Iodoform)	Silicone Oil	PEG	Xantangum	Lanolin	Others	Sum
**A**	**D30**	1	1	1					3
		33.33	33.33	33.33					100
	**Oil up-1**	1	1	1					3.5
		28.57	28.57	42.86					100
	**Oil up-2**	1	1	2					4
		25	25	50					100
	**Low Ca(OH)_2_**	0.5	1.5	1					3
		16.67	50	33.33					100
	**Low NaI**	1.5	0.5	1					3
		50	16.67	33.33					100
**B**	**PEG**	0.95	0.95	1.9	0.2				4
		23.75	23.75	47.5	5				100
	**Xantangum**	0.95	0.95	1.9		0.2			4
		23.75	23.75	47.5		5			100
	**Lanolin**	0.95	0.95	1.9			0.2		4
		23.75	23.75	47.5			5		100
**C**	**Lanolin**	0.95	0.95	1.9			0.2		4
		23.75	23.75	47.5			5		100
	**Oil down-1**	1.05	1.05	1.7			0.2		4
		26.25	26.25	42.5			5		100
	**Oil down-2**	1.15	1.15	1.5			0.2		4
		28.75	28.75	37.5			5		100
	**Oil down-3**	1.25	1.25	1.3			0.2		4
		31.25	31.25	32.5			5		100
**D**	**L 0**	1.15	1.15	1.7					4
		28.75	28.75	42.5					100
	**L 2.5**	1.15	1.15	1.6			0.1		4
		28.75	28.75	40			2.5		100
	**L 5**	1.15	1.15	1.5			0.2		4
		28.75	28.75	37.5			5		100
	**L 7.5**	1.15	1.15	1.4			0.3		4
		28.75	28.75	35			7.5		100
	**Vitapex**	30.3	40.4	22.4				6.9	100

## Data Availability

The original contributions presented in the study are included in the article, further inquiries can be directed to the corresponding author(s).
